# Modified Collard *versus* end‐to‐side hand‐sewn anastomosis for cervical anastomosis after McKeown esophagectomy

**DOI:** 10.1111/1759-7714.13630

**Published:** 2020-08-24

**Authors:** Xiao‐Kun Li, Yang Xu, Zhuang‐Zhuang Cong, Jing Luo, Hai Zhou, Sai‐Guang Ji, Yi‐Fei Diao, Wen‐Jie Wu, Yong Qiang, Jian‐Jun Qian, Yi Shen

**Affiliations:** ^1^ Department of Cardiothoracic Surgery, Jingling Hospital, School of Medicine Southeast University Nanjing China; ^2^ Department of Cardiothoracic Surgery, Jingling Hospital Medical School of Nanjing University Nanjing China; ^3^ Department of Cardiothoracic Surgery, Jingling Hospital, School of Clinical Medicine Nanjing Medical University Nanjing China; ^4^ Department of Clinical Medicine, School of Medicine Southeast University Nanjing China; ^5^ Department of Thoracic Surgery, Nanjing Second Hospital, School of Medicine Southeast University Nanjing China

**Keywords:** Anastomotic leakage, anastomotic stricture, end‐to‐side hand‐sewn anastomosis, esophagecotmy, modified Collard anastomosis

## Abstract

**Background:**

According to previously published studies, esophagectomy with modified Collard anastomosis has been reported to have low incidences of anastomotic leak and stricture. However, the optional anastomotic method after esophagectomy is still controversial. We conducted this study to compare the incidence of postoperative anastomotic stricture formation and dysphagia over three years after an esophagectomy with modified Collard anastomosis (MC) or end‐to‐side (ETS) hand‐sewn anastomosis. Meanwhile, the early postoperative anastomotic leakage and other complications, hospital stay and 30‐ and 90‐day mortality were also evaluated.

**Methods:**

The clinical data of 905 patients undergoing McKeown esophagectomy were retrospectively reviewed. The rate of postoperative stricture formation after three years was demonstrated by stricture‐free survival which is the primary end‐point of this study. The incidence of dysphagia, first time of onset of stricture and number of dilatations were also recorded during follow‐up.

**Results:**

The incidence of anastomotic leak tended to be higher in the MC group compared with that in the ETS group (13.0% vs. 8.7%, *P* = 0.064). The rates of anastomotic stricture in the MC group were significantly less than in the ETS group (*P* = 0.004). The number of dilatations in the MC group were significantly greater than those in the ETS group (2.34 vs*.* 2.46, *P* = 0.011).

**Conclusions:**

A modified Collard cervical esophagogastric anastomosis was associated with lower rates of anastomotic stricture and dysphagia, compared with ETS hand‐sewn anastomosis. However, the modified Collard anastomosis is accompanied by an increased anastomotic leakage rate.

## Graphical abstract

We conducted this study to compare the incidence of postoperative anastomotic stricture formation and dysphagia three years after an esophagectomy with modified Collard anastomosis or end‐to‐side (ETS) hand‐sewn anastomosis.

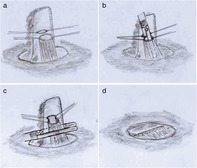

## Introduction

Esophageal cancer is the sixth most common cause of death and the eighth most common cancer worldwide.^1^ The prognosis for patients with esophageal cancer remains poor, with a five‐year overall survival rate of about 15%–34%.^2^ Radical esophagectomy with lymphadenectomy is still considered as the preferred therapeutic option for resectable esophageal cancer. However, the incidence of postoperative complications following this surgery which represents the most invasive procedure is reported to range widely from 45% to 80%.^3^, ^4^ The major concerns of postoperative complications are still anastomotic complications, including anastomotic stricture and leak. In many patients, overall survival is closely associated with a successful anastomosis and the avoidance of anastomotic tension and maintaining the blood supply to the tip of the gastric conduit, which may be impacted by the type of anastomosis, is critical. High tension and poor blood supply to the tip of the gastric conduit could result in high rate of anastomotic leakage. Patients with anastomotic leakage have three times the risk of death as those without leakage, and the mortality rate of the former can reach up to 60%. Meanwhile, preventing late benign stricture is also important due to it restoring patients' normal swallowing function.

The anastomotic technique during esophagectomy has been classified according to its type, location (neck or intrathoracic) and suturing (mechanical or hand‐sewn).^5^ End‐to‐end cervical anastomosis has been widely used for esophageal reconstruction. In some centers, however, end‐to‐side (ETS) hand‐sewn anastomosis has been reported to be superior to end‐to‐end anastomosis in terms of the rate of postoperative anastomotic leak and stricture formation.^6^ Collard *et al*.^7^ first reported the side‐to‐side anastomosis using a linear stapler which was then modified by Orringer *et al.*.^8^ The modified Collard (MC) method is considered to have superiorities in reducing postoperative anastomotic complications. According to previously published studies, esophagectomy with MC anastomosis has been reported to have low rates of anastomotic leak and stenosis.^9^ However, the optional anastomotic method after esophagectomy is still controversial. We conducted this study to compare the incidences of postoperative stricture formation and dysphagia after three years between a modified Collard anastomosis and end‐to‐side hand‐sewn anastomosis. The early postoperative anastomotic leakage and other complications, hospital stay and 30‐day and 90‐day mortality were also evaluated.

## Methods

The clinical data of 905 patients undergoing McKeown esophagectomy were retrospectively reviewed between 1 January 2014 and 1 January 2017 at the Department of Cardiothoracic Surgery in Jinling Hospital. The clinical data were collected from the medical records of patients. The inclusion criteria were: (i) Patients were pathologically diagnosed with esophageal cancer; (ii) patients had received McKeown esophagectomy with cervical MC or ETS anastomosis; and (iii) patients had undergone extended two‐field lymphadenectomies. The exclusion criteria were as follows: (i) Patient records lacked sufficient information for analysis; and (ii) patients had a history of other malignant tumors. There were 446 patients who had undergone MC and 459 patients who had undergone ETS during esophagectomy. Both groups had the same management and patient selection protocols. Our study was approved by the Ethics Committee of Jingling Hospital. The need for informed consents from the patients were waived by the ethics committee since this study was a retrospective cohort analysis and analyzed anonymously. The basic characteristics of patients included age, gender, BMI (body mass index), preoperative adjuvant therapy, smoking history, drinking history and comorbidity. Tumor‐related indicators included tumor location (upper, middle or lower), tumor size, histologic cell type, differentiation of the tumor (high, moderate or low), and pathological stage according to seventh Union for International Cancer Control (UICC). Surgical data was composed of surgical type (open operation, video‐assisted thoracoscopic surgery [VATS] or robotic‐assisted thoracic surgery [RATS]), surgical time, estimated blood loss (mL) supplementary enteral nutrition method (jejunostomy or duodenal nutrition tube) and the length of hospital stay. Early postoperative complications included chylothorax, pulmonary complications, anastomotic leakage and surgical site infection. The rate of postoperative stricture formation after three years was demonstrated by stricture‐free survival. The incidence of dysphagia, the first time of onset of stricture and the number of dilatations were also collected during follow‐up.

A contrast swallow study was performed at postoperative day 6 and endoscopy was conducted at day 7 after surgery. Contrast swallow studies were performed using water‐soluble contrast media. Normal oral intake was allowed after confirmation of the integrity of anastomosis on either investigation modalities. All patients were followed‐up every three months for the first three years, and then every six months thereafter. Our follow‐ups were implemented through outpatient department visit, home visit or telephone and the last follow‐up was conducted on 1 January 2020. The image examination during the follow‐up included chest computed tomography (CT) scan or videoendoscopy.

## Surgical procedure

Before surgery, the individual anastomotic approach was determined by patient choice after surgeons outlined the two anastomotic techniques to patients. All the operative and anastomotic procedures were completed by the same surgical group. Two‐field dissection was performed using RATS or VATS. The mediastinum was dissected from the diaphragm to the apex of the chest. The thorax was then closed with the patient placed in a supine position. In the abdominal portion, the right gastric and right gastroepiploic arteries were preserved to provide a vascular supply to the gastric conduit. During VATS and RATS lymphadenectomy, right and left RLN lymph nodes as well as #2R, #3P, #4R, #7, #8, #9 and #15 lymph nodes were carefully dissected in the thoracic phase in all patients. In addition, lymph nodes that were harvested in the abdominal phase included #16, #17, #18, #19 and #20 stations. A linear stapling device was used to create the gastric conduit with an incision between 3 to 4 cm along the greater curvature of the stomach. The staple line was oversewn with an additional PDS 3–0 suture. To expose the operative field, the patient was laid in a supine position with the neck extended at the very beginning and the head turned to the right afterwards. An oblique incision was made through the skin and muscles on the left side of the neck, and the prepared gastric conduit was gently pulled up with the specimen through the posterior mediastinum and removed to the neck. Two‐field dissection was performed in the open operation. The open operation started with a left thoracotomy, after which the esophagus was divided gently from the diaphragm to the apex of the chest. The stomach was explored through an incision in the left diaphragm and a gastric conduit was created, followed by the same VATS and the RATS procedures. During the lymphadenectomy in the open operation, #7, #8, #9 and #15 lymph nodes were carefully dissected in the thoracic phase in all patients. In addition, lymph nodes that were harvested in the abdominal phase included #16, #17, #18, #19 and #20 stations.

In end‐to‐side hand‐sewn anastomosis, the anastomosis was created at the posterior wall of the stomach tube, about 2 to 8 cm from the distal end of the gastric tube and with a diameter of 30–35 mm. This distance was determined by the approximate tension of the gastric conduit felt by the surgeon when pulling up the gastric tube. After completion of the ETS anastomosis, the tip of the gastric conduit was removed by stapling. Surgeons should anastomose to a certain position to remove as much of the redundant portion that is most ischemic as possible. Meanwhile the tension of the conduit cannot be too high. An additional PDS 3–0 suture was used to oversew the staple line.

**Figure 1:**
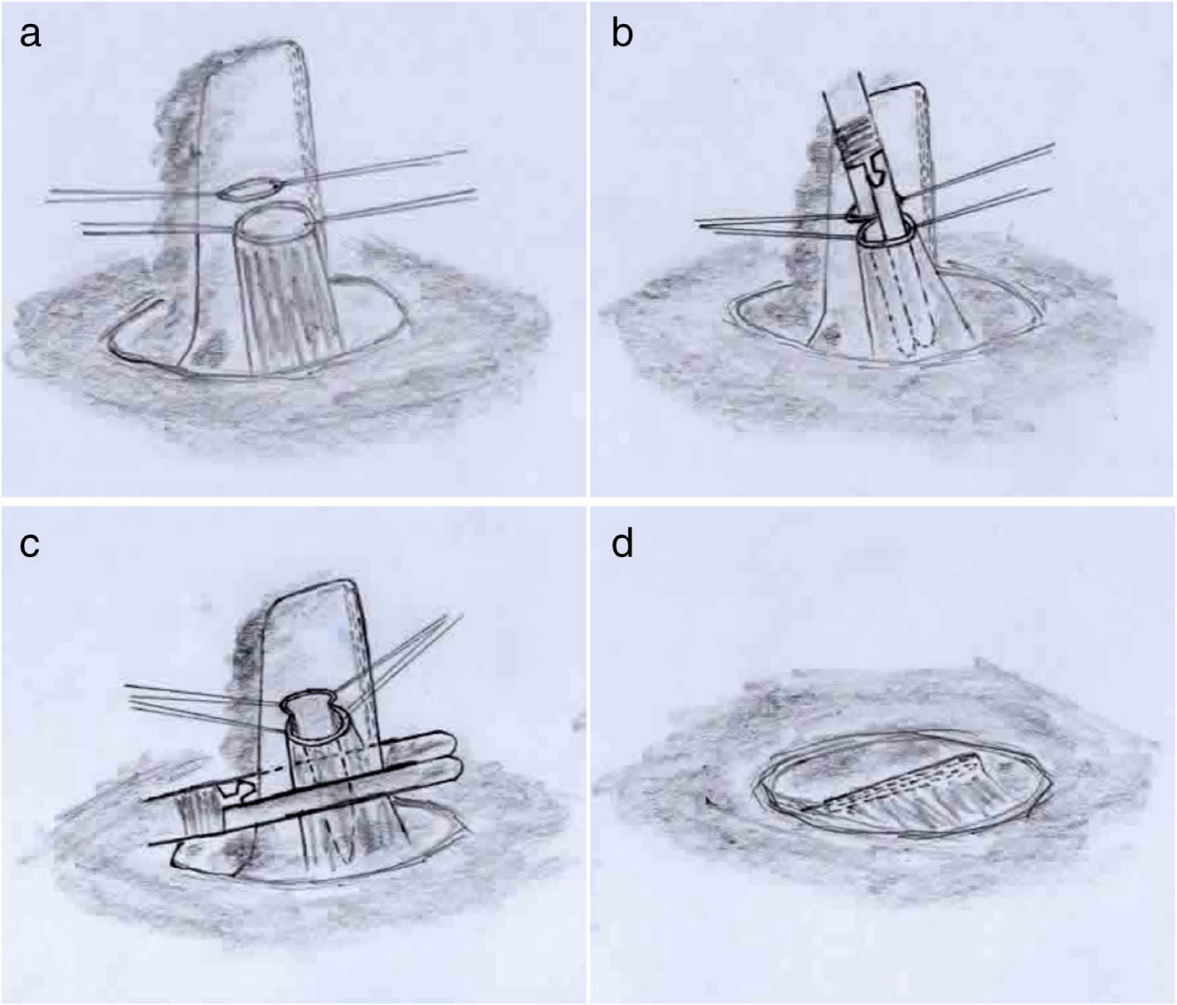
The procedure of modified Collard anastomosis.

In modified Collard anastomosis, the cervical esophagus was pulled out together with the gastric tube through the cervical incision. The proximal gastric conduit which has a poor blood flow was resected about 5 cm from the top of the gastric tube. On the posterior wall of the esophagus and gastric tube the stay suture was done. The posterior wall on the anastomosis was constructed by using a linear cutting stapler, with 45 mm insertion length of the staple in the anastomosis. Then, after the stay suture of the anterior wall of the esophagus and gastric tube was placed, a linear stapler was used to close the anterior wall. The seromuscular suture was added on the anterior wall, and then the anastomosis was replaced orthotopically (Fig 1 and Video S1).

### Definition of anastomotic leakage and stricture

Anastomotic leakage was defined as follows: (i) leaks confirmed by endoscopy, chest computed tomography (CT), and/or surgical exploration;^17^ and (ii) disruption of the anastomosis that leads to the leakage of the intraluminal content sufficient to cause clinical symptoms.

Anastomotic stricture was defined as dysphagia with barium esophagram or endoscopic proof of a stenosis. An 8.8 mm endoscope cannot be passed through the stenosis in the absence of recurrent esophageal cancer. The number of dilatations needed to relieve dysphagia was used to grade the severity of anastomotic stricture. “Severe stricture” was deemed as patients undergoing three or more dilations.

Dysphagia was graded on a five‐point scale from 0 to 4 according to the Mellow‐Pinkas‐score.^10^ A score of 4 was interpreted as a complete inability to swallow anything, and 0 was defined as no clinical dysphagia. Only the grade II dysphagia or worse were reported in this study. Severe dysphagia included patients with a score of 3 or more.

## Statistical analysis

Chi‐square or Fisher's exact test was used to compare categorical variables expressed as frequencies. The independent sample Student's *t*‐test or the MannWhitney nonparametric U test was used to compare continuous variables expressed as mean ± standard deviation. *P* < 0.05 was considered statistically significant. Statistical program SPSS 21.0 software (SPSS, Chicago, IL) was employed to analyze the data.

## Results

The perioperative and investigative profile of the entire study population is presented in Table 1. When comparing the patients' basic characteristics between two groups, there were no significant differences. Surgical data and short‐term clinical outcomes are also compared in Table 2. The incidence of anastomotic leakage tended to be higher in the MC group compared with that in the ETS group (13.0% vs. 8.7%, *P* = 0.064). No significant differences were found in 30‐day mortality and 90‐day mortality between the two groups (*P* = 0.33 and *P* = 0.38, respectively). The rate of postoperative stricture formation and dysphagia after three years were also compared (Table 3). The rates of anastomotic stricture (benign stricture) in the MC group were significantly less than in the ETS group (*P* = 0.004). Meanwhile, MC yielded a significantly lower rate of severe stricture compared with ETS (*P* = 0.024). However, there was no significant difference between the two groups in the rate of mild stricture (*P* = 0.150). The rate of dysphagia (≥ grade 2) in the MC group was significantly lower than that in the ETS group (*P* = 0.032). The MC anastomosis tended to yield lower rates of severe dysphagia (≥ grade 3) compared with ETS anastomosis (*P* = 0.084). The first time of onset of stricture in the MC group was significantly longer than that in the ETS group (*P* = 0.038). A significantly higher number of dilatations in the ETS group were found compared with the MC group (*P* = 0.011).

**Table 1:** Basic characteristics

	MC (446)	ETS (459)	*P*‐value
Age	64.26 ± 8.36	64.40 ± 8.31	0.8
Gender			0.38
Male	349 (78.3%)	347 (75.6%)	
Female	97 (21.7%)	112 (24.4%)	
BMI (kg/m^2^)	23.19 ± 3.24	23.20 ± 3.27	0.94
smoking history	235 (52.7%)	227 (49.5%)	0.35
Drinking history	190 (42.6%)	194 (42.3%)	0.95
Diabetes	31 (7.0%)	32 (7.0%)	1
Hypertension	128 (28.7%)	133 (29.0%)	0.94
Cardiovascular complication	176 (39.5%)	178 (38.8%)	0.84
Respiratory complication	58 (13.0%)	50 (10.9%)	0.36
COPD	42 (9.4%)	38 (8.3%)	0.56
Tumor size	3.43 ± 1.57	3.52 ± 1.67	0.43
Tumor location			0.56
Upper	35 (7.8%)	42 (9.2%)	
Middle	266 (59.6%)	281 (61.2%)	
Lower	145 (32.5%)	136 (29.6%)	
Histological type			0.85
SCC	413 (92.6%)	430 (93.7%)	
AC	5 (1.1%)	6 (1.3%)	
ASC	2 (0.4%)	2 (0.4)	
Others	26 (5.9%)	21 (42.9%)	
Pathologic tumor stage			0.84
I	175 (39.2%)	186 (40.5%)	
II	96 (21.5%)	106 (23.1%)	
III	162 (36.3%)	154 (33.6%)	
IV	13 (2.9%)	13 (2.8%)	
Differentiation of the tumor			0.58
High	124 (27.8%)	136 (29.6%)	
Moderate	244 (54.7%)	235 (51.2%)	
Low	78 (17.5%)	88 (19.2%)	
Preoperative adjuvant therapy	67 (15.0%)	81 (17.6%)	

BMI, body mass index; COPD, chronic obstructive pulmonary disease; SCC, squamous cell carcinoma; AC, adenocarcinoma; ASC, adeno‐squamous carcinoma.

**Table 2:** Surgical data and short‐term clinical outcomes

	MC (446)	ETS (459)	*P*‐value
Surgical type			0.53
Open operation	192 (43.0%)	208 (45.3%)	
VATS	190 (42.6%)	179 (39.0%)	
RATS	64 (14.3%)	72 (15.7%)	
Surgical time (hours)	4.03 ± 1.11	4.00 ± 1.10	0.72
Estimated blood loss (mL)	107.4 ± 65.4	109.1 ± 71.2	
Enteral nutrition approach			0.47
Jejunostomy	90 (20.0%)	84 (18.3%)	
Duodenal nutrition tube	356 (79.8%)	375 (81.7%)	
Chylothrax	6 (1.3%)	6 (1.3%)	0.96
Surgical site infection	29 (6.5%)	31 (6.8%)	0.55
Pulmonary complication	23 (5.2%)	19 (4.1%)	0.47
Pneumonia	18 (4.0%)	14 (3.1%)	0.42
Anastomotic leakage	58 (13.0%)	40 (8.7%)	0.064
Hospital stay	19.78 ± 17.10	19.10 ± 17.25	0.55
30‐day mortality	12 (2.7%)	8 (1.7%)	0.33
90‐day mortality	15 (3.4%)	11 (2.4%)	0.38

VATS, video‐assisted thoracoscopic surgery; RATS, robotic‐assisted thoracic surgery.

**Table 3:** Long‐term anastomotic complications (in three years)

	MC (446)	ETS (459)	*P*‐value
Anastomotic stricture (benign stricture)	84 (11.9%)	124 (17.2%)	0.004
Mild stricture (1–2 sessions)	31 (7.0%)	45 (9.8%)	0.150
Severe stricture (≥ 3 sessions)	53 (11.9%)	79 (17.2%)	0.024
Dysphagia ≥ grade 2 (Mellow‐Pinkas‐score)	89 (20.0%)	131 (28.5%)	0.032
Severe dysphagia ≥ grade 3 (Mellow‐Pinkas‐score)	56 (12.6%)	83 (18.1%)	0.084
Onset of stricture (months)	4.80 ± 2.33	4.06 ± 1.87	0.038
Number of dilatation	2.34 ± 0.51	2.46 ± 0.82	0.011

## Discussion

Anastomotic stricture and leakage are still the main causes of esophagogastric anastomotic failure after esophagectomy, which has stimulated various anastomotic methods.^11^, ^12^, ^13^ The best procedure to conduct this anastomosis is still controversial.

In some studies, the end‐to‐end hand‐sewn esophagogastric anastomosis was considered as the preferred technique, compared with different anastomotic techniques, with 4%–25% anastomotic leaks and 9%–45% anastomotic stricture.^14^, ^15^, ^16^, ^17^, ^18^ In some centers, however, end‐to‐side anastomosis proved superior to end‐to‐end anastomosis with regard to the rate of postoperative anastomotic leakage and stricture formation.^19^

Cervical side‐to‐side anastomosis was first reported by Collard *et al*. with an anastomotic leakage rate of 6%.^7^ Ercan *et al*. subsequently performed modified Collard anastomosis after esophagectomy and noted that the rate of anastomotic leakage was 4%.^20^ Prince *et al*. also conducted this anastomotic technique with an anastomotic leakage incidence of 21%.^21^ The incidence of anastomotic leakage of MC in our study was acceptable (13.0%), compared with end‐to‐side hand‐sewn anastomosis with an incidence of 8.7%. Given our results, MC yields a higher rate of anastomotic leak which is opposite to that reported by Sugimura *et al*. in their study which is the first report comparing the short‐term clinical outcomes between the MC technique and other anastomotic techniques.^9^ The results of their study revealed that the incidence of anastomotic leakage in the MC group tended to be lower than that in the hand‐sewn group; however, the difference was not statistically significant.

The results of the present study reported that modified Collard anastomosis yielded significantly lower rates of anastomotic stricture compared with hand‐sewn anastomosis. In this study, the ETS group yielded an anastomotic stricture rate of 27.0%. The stricture rate of hand‐sewn anastomoses reported in the review by Deng *et al*. was 54%.^5^ Another review reported by Honda *et al*. showed that the stricture rate of hand‐sewn anastomoses was 10%.^6^ A potential explanation for the high incidence of anastomotic stricture might be due to the use of a double‐layer suture and the small size of the anastomotic site. Meanwhile, the resection length in the proximal gastric tube which was most ischemic was too short in the hand‐sewn group, which could be another reason for these differences. Ischemia around the anastomotic site could also result in anastomotic stricture. Given the previous studies regarding the modified Collard anastomosis, the rate of anastomotic stricture ranged widely from 4.6% to 66%. According to the study by Prince *et al*. the rate of anastomotic stricture after MC was 24%, ^21^ and according to another study reported by Ercan *et al*. the incidence of anastomotic stricture was 66%.^20^ Sugimura *et al*. showed that MC yielded a rate of stricture of 22%,^9^ whereas the study by Behzadi *et al*. revealed that the rate of stricture after modified Collard anastomosis was 4.6%,^22^ which was lower compared with that in our study (18.8%). The insertion length of the staple in the anastomosis of the posterior wall could be the potential reason for this difference. One of the superiorities of the modified Collard anastomosis is its large anastomotic diameter. The study of Behzadi *et al*.^22^ reported an insertion length of 45–50 mm resulting in a low stenosis rate and Sugimura *et al*.^9^ reported a 40 mm insertion length of the staple in the anastomosis. The insertion length in our study was 45 mm. Technically, the longer the staple that is inserted, the larger the diameter of the anastomosis that can be formed, which could logically decrease the stricture rate. However, the insertion length cannot be too long since the blood flow of the proximal gastric tube may become worse and the tension in the suture of the posterior wall could increase. Therefore, more studies are needed to investigate the appropriate insertion length.

In this study, anastomotic stricture formation instead of leakage was selected as the primary endpoint since it defeats one of the vital aims of esophagectomy, which is to restore normal swallowing function. The quality of life in patients undergoing esophagectomy was negatively affected by dysphagia which occurs in up to 66% of the patients in the postoperative period.^20^, ^23^, ^24^ The anastomotic stricture is the main cause of dysphagia. About 70% patients with dysphagia grade 2 or worse were found and subsequently proven to have a stricture following endoscopy. The rate of anastomotic strictures in this study seems higher than that in the study reported by Collard *et al*. (6.3%).^7^ However, follow‐up in our study was over a three year period and much longer than that of their study (60 days). Significant differences were found between the two groups in the number of dilatation sessions needed to relieve symptoms and the first time to onset of stricture (first dilatation session), when stricture formation occurred. The first time of stricture formation after surgery in the MC group (4.80 ± 2.33 months) was significantly longer than that (4.06 ± 1.87 months) in the ETS group. An anastomotic stricture occurred in 10 of the 58 patients with anastomotic leakage in the MC group and 16 of the 40 patients who had anastomotic leakage in the ETS group. These results differ from the results of Briel *et al*. which reported that anastomotic leakage was an independent risk factor for stricture.^25^ The high rate of stricture and dysphagia in the ETS group could severely influence the oral nutritional supplements of the patients undergoing esophagectomy. Furthermore, the suboptimal intake of nutrients could cause continuous weight loss which is considered as an independent risk factor for oncological prognosis.^26^, ^27^, ^28^

According to the results, we found a high rate of anastomotic leakages in the MC group. One of the technical reasons for the high leakage rate could be due to resection of the tips of both the gastric conduit and esophagus which could cause more anastomotic tension. Another reason could be because of the uneven tension in the sutures. The posterior wall of the esophagus and the gastric conduit, which was considered as the most tensive place, was first constructed using a linear cutting stapler and no sutures were added. However, the anterior wall was closed using the linear stapler and the seromuscular suture of the anterior wall was added which made the sutures firmer than those in the posterior wall where leaks were most commonly found via endoscopy. Meanwhile, in clinical practice, oversewing the staple line may contribute to additional ischemia, leading to dehiscence of the suture and staple line. This could be another potential reason for the increased leakage rate in the MC group.

There were several limitations in our study. First, this was a single‐center retrospective study and selection bias was unavoidable due to its retrospective nature. Second, there were many risk factors resulting in anastomotic complications. Although, we only evaluated the patients undergoing McKeown esophagectomy with cervical anastomosis and two‐field lymphadenectomy, the bias between the two groups could not be totally eliminated.

In conclusion, a modified Collard cervical esophagogastric anastomosis in the neck is associated with a lower anastomotic stricture and dysphagia rate, compared with end‐to‐side hand‐sewn anastomosis. However, the modified Collard anastomosis is accompanied by an increased anastomotic leakage rate.

## Disclosure

The authors declare that there are no conflicts of interest.

## Supporting information


**Video S1.** The procedure of modified Collard anastomosis.
